# Specificity and off-target effects of AAV8-TBG viral vectors for the manipulation of hepatocellular gene expression in mice

**DOI:** 10.1242/bio.058678

**Published:** 2021-09-22

**Authors:** Christos Kiourtis, Ania Wilczynska, Colin Nixon, William Clark, Stephanie May, Thomas G. Bird

**Affiliations:** 1 Cancer Research UK Beatson Institute, Glasgow G61 1BD, UK; 2 Institute of Cancer Sciences, University of Glasgow, Glasgow G61 1QH, United Kingdom; 3 MRC Centre for Inflammation Research, The Queen's Medical Research Institute, University of Edinburgh, Edinburgh EH164TJ, UK

**Keywords:** Adeno-associated virus, Liver disease, Mouse model, Genetic models

## Abstract

Mice are a widely used pre-clinical model system in large part due to their potential for genetic manipulation. The ability to manipulate gene expression in specific cells under temporal control is a powerful experimental tool. The liver is central to metabolic homeostasis and a site of many diseases, making the targeting of hepatocytes attractive. Adeno-associated virus 8 (AAV8) vectors are valuable instruments for the manipulation of hepatocellular gene expression. However, their off-target effects in mice have not been thoroughly explored. Here, we sought to identify the short-term off-target effects of AAV8 administration in mice. To do this, we injected C57BL/6J wild-type mice with either recombinant AAV8 vectors expressing Cre recombinase or control AAV8 vectors and characterised the changes in general health and in liver physiology, histology and transcriptomics compared to uninjected controls. We observed an acute and transient trend for reduction in homeostatic liver proliferation together with induction of the DNA damage marker γH2AX following AAV8 administration. The latter was enhanced upon Cre recombinase expression by the vector. Furthermore, we observed transcriptional changes in genes involved in circadian rhythm and response to infection. Notably, there were no additional transcriptomic changes upon expression of Cre recombinase by the AAV8 vector. Overall, there was no evidence of liver injury, and only mild T-cell infiltration was observed 14 days following AAV8 infection. These data advance the technique of hepatocellular genome editing through Cre-Lox recombination using Cre expressing AAV vectors, demonstrating their minimal effects on murine physiology and highlight the more subtle off target effects of these systems.

## Abstract (precis)

**Summary:** This paper provides a comprehensive characterisation of the short-term effects of administration of adeno-associated virus 8 on murine physiology, liver histology and liver transcriptome.

## INTRODUCTION

Animal models have improved our understanding and therapies for human disease. The mouse is a prototypical model organism that is widely used for a number of reasons, including its similarities with human physiology, breeding efficiency and ease of handling, cost efficiency and the range of available genetic models. Due to the latter particularly, mice have become the most widely used *in vivo* pre-clinical model system (Rosenthal and Brown, 2007). Manipulation of gene expression in this model organism has come a long way from whole body knockout (KO) to the current point that we are able to introduce point mutations in a tissue specific manner through CRISPR-Cas9 genomic editing (Sauer and Henderson, 1988; Wilson, 1996; Lee et al., 2020a; Lundin et al., 2020). The Cre-Lox system, although less flexible compared to CRISPR, remains widely used for the manipulation of gene expression in mice and is a readily applicable means of genomic editing with high reproducibility.

Taking advantage of the Cre-Lox system, Adeno-associated viruses (AAVs) are an important vector system for gene expression manipulation and their use has risen dramatically in the last 20 years. As AAVs are replication deficient, they are a relatively safe and efficient way to express the Cre recombinase, overexpress specific proteins or introduce shRNA into *in vivo* model systems. AAVs are small (20 nm), single-stranded DNA viruses that belong to the family of Parvoviridae. They elicit a very mild immune response, especially the recombinant AAV vectors (rAAVs) that have undergone modifications to partly evade the immune system (Rogers et al., 2011; Rabinowitz et al., 2019). There are different serotypes of AAV (AAV1, 2, 4, 5, 6, 7, 8, and 9), each of which exhibits a various transduction efficiencies in the different target tissues (Zincarelli et al., 2008). In mice, after transducing their target cells, AAVs enter the cell nucleus where they persist in an episomal form and only rarely integrate into the host genome (Duan et al., 1999; Miller et al., 2004).

The liver is the largest solid organ in the body and is a frequent site of organ-specific and systemic diseases and a common site of tumour metastasis. In liver biology, studying hepatocytes is particularly important as they constitute the majority of liver cells, comprising around 80% of total liver mass. Hepatocytes perform most of the synthetic and detoxification functions of the liver, are major contributors to liver regeneration and are the cell of origin for the majority of primary liver cancers (Müller et al., 2020). As a result, genetic manipulation of hepatocytes is a powerful tool in the study of liver disease.

There are a number of ways to manipulate hepatocellular gene expression (Kellendonk et al., 2000). Currently, a widely used approach is to target hepatocytes with an AAV-based vector. rAAV8 is a commonly used AAV serotype due to its strong propensity to transduce hepatocytes (Nakai et al., 2005). rAAV8-mediated hepatocellular gene editing has multiple applications including gene therapy (Nathwani et al., 2011), lineage tracing experiments, gene deletion or gene overexpression in all or specific populations of the hepatocytes. Through the insertion of tissue-specific promoters, expression of the vector's ‘cargo’ can be further cell type-restricted. In particular, the Cre recombinase together with a hepatocyte-specific promoter like the Thyroxin Binding Globulin (*TBG*) promoter can be incorporated into the AAV8 genome and this is reported to be a specific means of Cre recombinase expression in hepatocytes, while avoiding undesired expression in extrahepatic cells (Nakai et al., 2005; Malato et al., 2011; Lee et al., 2020a,b). The number of transduced hepatocytes is proportional to the dose (i.e. genetic copies) of AAV8-*TBG* vector that are administered; the higher the dose of the vector, the more hepatocytes will be transduced. This allows the study of deleting/overexpressing a gene in the whole liver parenchyma (Bird et al., 2018) or in a small number of hepatocytes using comparatively fewer genetic copies of vector. Alternatively, instead of the Cre recombinase, it is possible to deliver other constructs as cargo (e.g. expression of shRNAs or ectopic proteins) to hepatocytes using this approach; for example, administration of the AAV8-*TBG-*P21 vector results in P21 overexpression in hepatocytes, inhibiting their ability to proliferate (Raven et al., 2017). Expression of ectopic proteins with AAV vectors has been reported to last for several months, at least in post-mitotic cells (Duan et al., 1999).

The AAV8 system theoretically allows for manipulation of gene expression at a desired time point and without inducing toxicity or the risk of genetic ‘leakiness’ through an endogenous Cre allele. This is in comparison to other models like the Albumin-Cre mice, where the Cre recombinase is constitutively expressed from embryonic life and is therefore not temporally controlled, or tamoxifen-mediated manipulation of gene expression, where tamoxifen has been reported to induce toxicity (Gao et al., 2016; Keeley et al., 2019). As such, AAV8-*TBG* is widely used in order to recombine the majority of the hepatocytes and study the effects of gene expression changes in the whole liver serving as a single hit, hepatocyte-specific gene knockout/overexpression.

With the report that AAVs may have long lasting effects upon the liver epithelium, including rare cancers, it is clear that transduction with AAV is not entirely benign (Nault et al., 2015). Even though in humans evidence suggests that the immune system might compromise AAV8 efficiency (partly due to cross-immunity with adenoviruses) there have not been detailed studies on the murine immune response against AAV8 (Boutin et al., 2010; Mendell et al., 2010; Calcedo et al., 2011). Furthermore, as rAAV8 rarely integrates into the murine host genome, it seems unlikely that it would cause significant genotoxicity. In one study investigating the long term effects of AAV2-hFIX16 (which results in liver-specific expression of clotting factor IX) in liver tumourigenesis in mice, it was found that there was no association between tissue from hepatocellular carcinomas (HCCs) and AAV copy numbers (Li, et al., 2011).

Transcriptome-wide studies are commonly performed on whole liver lysates or isolated liver cell fractions of mice treated with AAV8-*TBG*-Cre. These transcriptomics analyses can give valuable information on the effects following manipulation of hepatocellular gene expression via AAV8-*TBG*-Cre. However, a potential effect on the transcriptome by the AAV8 vector or by its cargo (i.e. the Cre recombinase or other protein expressed by the vector) should be taken into consideration when performing and interpreting such studies. To our knowledge there are currently no studies addressing whether AAV vectors (and in particular AAV8-*TBG*) alone have an effect on the liver transcriptome.

Overall, there is a lack of descriptive studies on the effects of systemic AAV8 administration in mice. Therefore, to address this shortfall we investigated the short-term off-target effects of systemic AAV8-*TBG* administration in wild-type (WT) mice. After intravenous (IV) injection of AAV8-*TBG*-Cre (expressing Cre recombinase) or AAV8-*TBG*-Null (expressing a scrambled sequence) at dosing resulting in transduction across the majority of the hepatocellular compartment we examined both liver specific and systemic alterations in WT mice. Using blood analysis combined with immunohistochemistry and transcriptomics analysis we describe the effects occurring over 2 weeks post transduction. These data confirm minor off target effects following transduction using this experimental strategy and serve as a reference tool for the research community.

## RESULTS

### AAV8-*TBG* is hepatocyte-specific

**Fig. 1.**
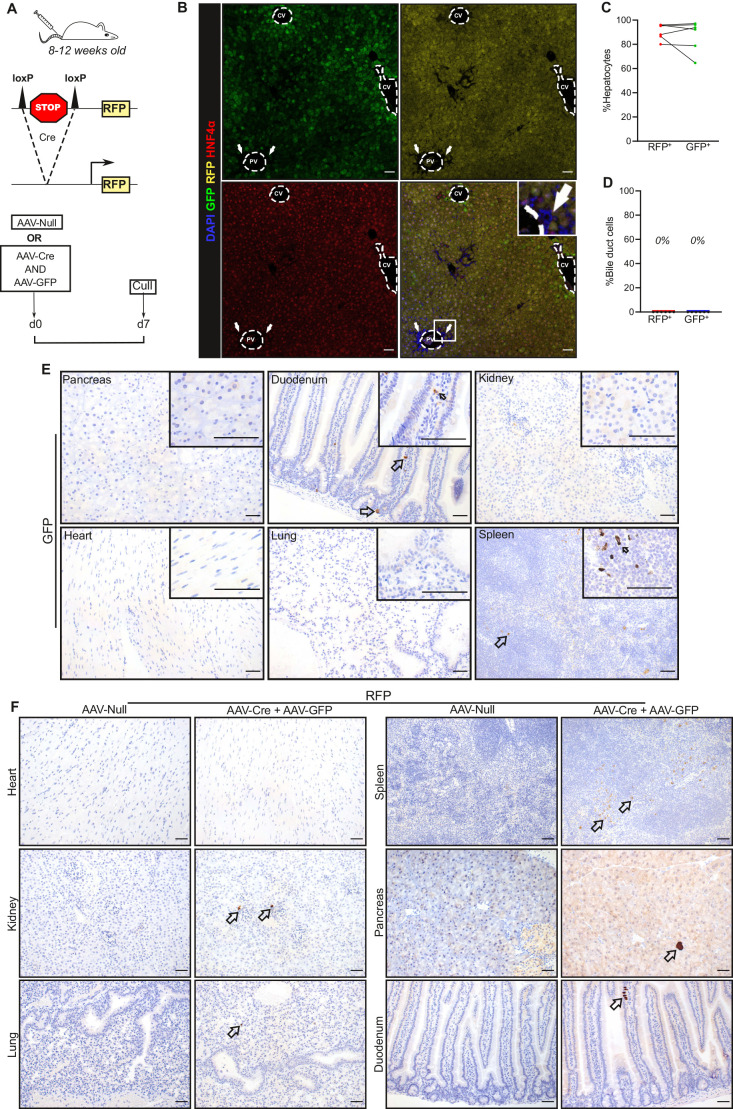
**AAV8-*TBG* vectors specifically target the hepatocytes.** (A) Schematic of the experimental design; 8–12 week old male LSL-RFP mice on a C57BL/6 background (*n*=6) were IV injected with AAV-Cre and AAV-GFP at the same dose (2×10^11^ GC/mouse). LSL-RFP mice (*n*=4) injected with AAV-Null served as controls. 7 days post injection their livers were harvested for analysis. (B) Representative images from liver sections stained for DAPI (blue), GFP (green), RFP (yellow) and the hepatocyte-specific marker HNF4α (magenta), showing the hepatocellular specificity of the AAV8-*TBG* vectors. Arrows highlight the unlabelled bile ducts. CV, central vein; PV, portal vein. (C) Quantification of GFP^+^ and RFP^+^ hepatocytes (i.e. HNF4a^+^ cells) in the livers of the six mice described in Fig. 1A and B, shown as percentage of total hepatocytes. (D) Quantification of RFP^+^ and GFP^+^ bile duct cells in the livers of the six mice described in Fig. 1A and B. (E) Representative images of GFP immunohistochemistry in the pancreas, duodenum, kidney, heart, lung and spleen of mice injected with AAV-Cre and AAV-GFP. The inset images are from GFP-stained liver sections from uninjected WT mice (i.e. mice not injected with either AAV-Cre or AAV-GFP, representative images from *n*=3 mice). Arrows highlight GFP^+^ cells. (F) Immunohistochemistry for RFP in the kidney, pancreas, spleen, heart, lung and duodenum of the mice described in Fig. 1A. Arrows highlight RFP^+^ cells. Scale bars: 50 μm.

We first examined the tissue and cell specificity of AAV8-*TBG* using mice homozygous for the R26-LSL-tdTomato allele on a C57BL/6 background by simultaneous injection with AAV8-*TBG*-Cre and AAV8-*TBG*-GFP (herein referred to as AAV-Cre and AAV-GFP, respectively) (Fig. 1A). The cells expressing the Green Fluorescent Protein (GFP) and Red Fluorescent Protein (RFP) reporters 7 days after AAV8 injection were assessed histologically first in the liver, demonstrating that the majority of the hepatocytes expressed the reporters (80–96% for RFP and 64–97% for GFP) (Fig. 1B,C; Fig. S1A,B), consistent with previous reports using this (Bird et al., 2018; Gay et al., 2019) and other AAV8-Cre constructs (Malato et al., 2011). There was no evidence of recombination of biliary epithelium (Fig. 1D). Interestingly, while RFP staining was distributed evenly across the hepatocytes, the GFP distribution was more irregular and its intensity varied among hepatocytes, with a tendency for more intense staining in the hepatocytes surrounding the central vein (pericentral hepatocytes of Zone 3) (Fig. 1B). Notably, when we checked for reporter expression in other organs, we observed labelling of very few cells in the duodenum, kidney, pancreas, lung and the spleen (Fig. 1E,F). The apparent GFP positivity observed in the duodenum and the spleen of uninjected mice (Fig. 1E, inset images) appears as non-specific background staining. These data show, in agreement with other studies (Wang et al., 2010; Bell et al., 2011b), that AAV8-*TBG*-mediated gene targeting is highly specific for hepatocytes with negligible targeting of extra-hepatic tissues.

### Systemic administration of AAV8-*TBG* does not affect the general health of mice

**Fig. 2.**
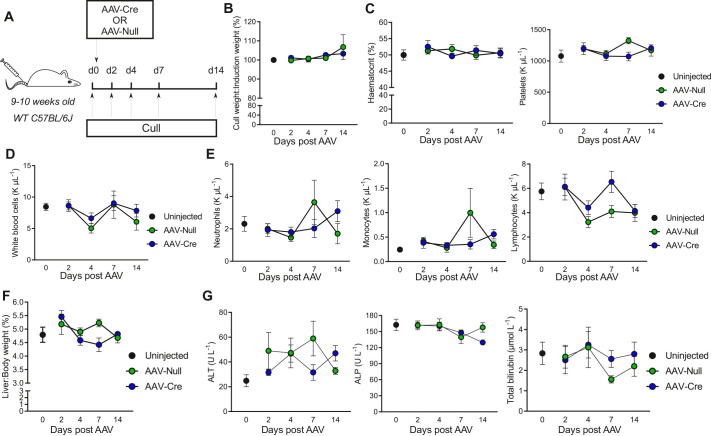
**Systemic administration of AAV8-*TBG* has minimal effects on general health causing neither liver injury nor impaired liver function.** (A) Schematic of experimental outline. Male C57BL/6J WT mice (*n*=56) were injected IV with either AAV-Null or AAV-Cre. Uninjected control mice (*n*=6) from the same stock were culled on the day that the rest of the mice were injected with AAV8-*TBG* (day 0). The injected mice were culled 2 (*n*=12; 6 AAV-Null and 6 AAV-Cre), 4 (*n*=16; 8 AAV-Null and 8 AAV-Cre), 7 (*n*=18; 9 AAV-Null and 9 AAV-Cre) or 14 (*n*=10; 5 AAV-Null and 5 AAV-Cre) days after injection. (B) Body weight at cull in relation to body weight at day 0 for the mice described in Fig. 2A. Kruskal–Wallis test showed no statistically significant differences. (C) Haematocrit and Platelet counts for the mice described in Fig. 2A. One-way ANOVA showed no statistically significant differences. (D) Circulating white blood cell counts for the mice described in Fig. 2A. Kruskal–Wallis test showed no statistically significant differences. (E) Absolute blood counts of circulating neutrophils, monocytes and lymphocytes for the mice described in Fig. 2A. Kruskal–Wallis test (for neutrophils and monocytes) or one-way ANOVA (for lymphocytes) showed no statistically significant differences. Data are mean±s.e.m. (F) Liver weight to body weight ratio for the mice described in Fig. 2A. Kruskal–Wallis test showed no statistically significant differences. (G) ALT, ALP and total bilirubin in the plasma of the mice described in Fig. 2A. Kruskal–Wallis test showed no statistically significant differences. All data on graphs are mean±s.e.m.

To investigate the off-target effects of systemic AAV8-*TBG* administration, WT mice were IV injected with AAV8-*TBG*-Null (herein referred to as AAV-Null) or AAV-Cre. Mice were then culled 2, 4, 7 or 14 days post AAV8-*TBG* injection and compared to uninjected controls using a number of clinical parameters (Fig. 2A). Starting at a similar body weight at day 0 (Fig. S1C), the mice showed no significant changes in body weight and gradually gained weight at a normal rate for their age during the 2 weeks following AAV-Null or AAV-Cre, regardless of the group (Fig. 2B). Haematology analysis showed no changes in haematocrit or platelets (Fig. 2C). Reflecting the reported mild inflammatory response elicited by AAVs, we did not observe significant changes in circulating total white blood cells, monocytes, neutrophils or lymphocytes (Fig. 2D,E; Fig. S1D). Overall, we did not observe any impact on general health of mice a week after AAV-Null or AAV-Cre administration.

### AAV8-*TBG* vectors do not cause liver damage

Next, having demonstrated hepatocyte-specific targeting, we proceeded to assess the effects of AAV8-*TBG* on the liver specifically. Livers were normal macroscopically and we did not observe any changes in liver size or liver histology microscopically (as assessed by H&E staining) in response to AAV8-*TBG* (Fig. 2F; Figs S1E and S2). Similarly, serum levels of alanine aminotransferase (ALT) and alkaline phosphatase (ALP) (markers of liver necrosis and bile duct damage, respectively) remained at baseline levels at every time point (Fig. 2G). Assessing liver function, serum bilirubin levels also remained unaffected as did serum levels of total protein and globulins (Fig. 2G; Fig. S1F). We noticed a significant increase in albumin:globulins ratio in the blood, which was driven in part by a significant increase in serum albumin but also by a trend for reduction of serum globulins (Fig. S1F). Examining hepatic cell death in more detail, we performed immunohistochemistry for the apoptosis-specific marker cleaved caspase 3 (CC3). No changes in apoptotic cell death were observed at any time point (Figs S1G and S2). There was no change in serum urea levels, however creatinine was significantly increased at day 4 and 14 in AAV-Null mice (Fig. S1H). Therefore, we found no evidence of liver damage and only observed mild dysfunction, as evidenced by the increase in serum albumin, after AAV8-*TBG* administration during the times when transduction and genetic recombination occur.

**Fig. 3.**
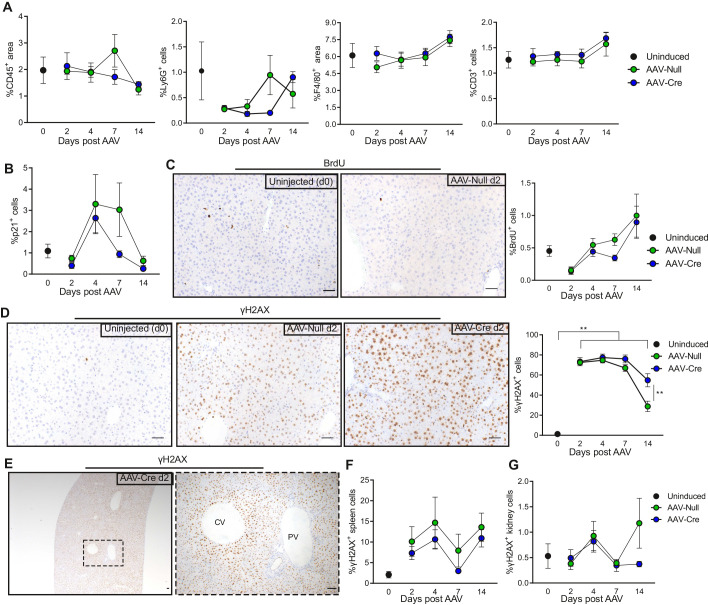
**AAV8-*TBG* vectors affect the hepatocellular cell cycle and result in DNA damage response.** (A) Quantification of hepatic CD45, Ly6G, F4/80 and CD3 based on positive area/total liver area (CD45, F4/80) or positive cells as a percentage of total cells (CD3, Ly6G) after immunohistochemical detection (representative images for each time point shown in Figs S2 and S3). Kruskal–Wallis test (for CD3 and Ly6G) or Brown-Forsythe and Welch ANOVA (for CD45 and F4/80) showed no statistically significant differences. (B) Quantification of liver P21^+^ cells presented after immunohistochemical detection (representative images for each time point in Fig. S3). Data are presented as percentage of total liver cells. Brown-Forsythe and Welch ANOVA showed no statistically significant differences. (C) Quantification of liver cells positive for the S-phase marker BrdU and representative immunohistochemistry images (additional images for each time point are shown in Fig. S3). (D) Quantification of γH2AX^+^ liver cells and representative immunohistochemistry images (additional images for each time point are shown in Fig. S4). Brown-Forsythe and Welch ANOVA was used for comparisons with the uninjected mice. An unpaired *t*-test was used for the day 14 time point (AAV-Null versus AAV-Cre). *P*=**<0.01. (E) Representative liver section stained for γH2AX showing zonal staining particularly in the pericentral area (Zone 3). CV, central vein; PV, portal vein. (F) Quantification of γH2AX^+^ spleen cells (representative images for each time point in Fig. S4). Brown-Forsythe and Welch ANOVA showed no statistically significant differences. (G) Quantification of γH2AX^+^ kidney cells (representative images for each time point in Fig. S4). Brown-Forsythe and Welch ANOVA showed no statistically significant differences. For all graphs *n*=4 in all groups apart from day 7 and day 14 time points where *n*=5 for each group. For each graph data are mean±s.e.m. and scale bars: 50 μm.

We next examined intrahepatic leukocyte populations to see whether a demonstrable local immune response occurred in the liver. Using the pan-leukocyte marker CD45, we did not observe any change in overall hepatic leukocyte numbers or distribution (Fig. 3A; Fig. S2). The use of more specific leukocyte markers for neutrophils (Ly6G), macrophages (F4/80) and T-cells (CD3) also demonstrated no significant differences in these populations either in number or distribution at any time point (Fig. 3A; Figs S2 and S3). Therefore we find no evidence of histological inflammation or inflammatory response to biologically relevant AAV8 dosing.

### AAV8-*TBG* vectors affect the cell cycle of liver cells and induce expression of the DNA damage marker γH2AX in the liver

Viral infection of mammalian cells is, through a variety of well characterised mechanisms, known to affect several cellular processes including cell cycle, DNA damage response (DDR) and the release of damage-associated molecular patterns (DAMPs) (Loo and Gale, 2011; Dou et al., 2017; Motwani et al., 2019). To address whether AAV8-*TBG* vectors can induce such changes, we first stained liver sections for the cell cycle inhibitor *Cdkn1a* (P21) or for BrdU to determine changes in the cell cycle status of liver cells. Whilst there was no significant change in hepatic P21 at any time point in either group, there was a trend for transient reduction of BrdU^+^ cells at day 2 post AAV8-*TBG* administration with a rebound at the 2 week time point (Fig. 3B,C; Fig. S3). Next, we assessed the presence and extent of hepatic DNA damage by staining liver sections for the DNA damage marker γH2AX. We observed a marked increase in γH2AX at day 2, persisting until day 7 and falling at day 14 more prominently in the AAV-Null compared to AAV-Cre (Fig. 3D; Fig. S4). Moreover, treatment with AAV-Cre resulted in a stronger γH2AX response in the liver (Fig. 3D; Fig. S1I). Notably, γH2AX staining was stronger in the pericentral hepatocytes (Fig. 3E). While gene expression through AAV8-TBG is highly liver specific, AAV8 transduction is less well restricted. Therefore, we investigated whether there was induction of γH2AX in other organs prone to AAV8 transduction. To do this we stained spleen and kidney sections for γH2AX. We observed no γH2AX induction in the kidney and a trend for induction in the spleen, particularly localised within the red pulp (Fig. 3H,I; Fig. S4) Overall, our data reveal an acute and transient reduction in hepatic proliferation alongside a temporally-associated increased hepatocellular γH2AX expression following systemic AAV8 administration.

### AAV8-*TBG* vectors induce circadian rhythm- and infection-related transcriptional changes

**Fig. 4.**
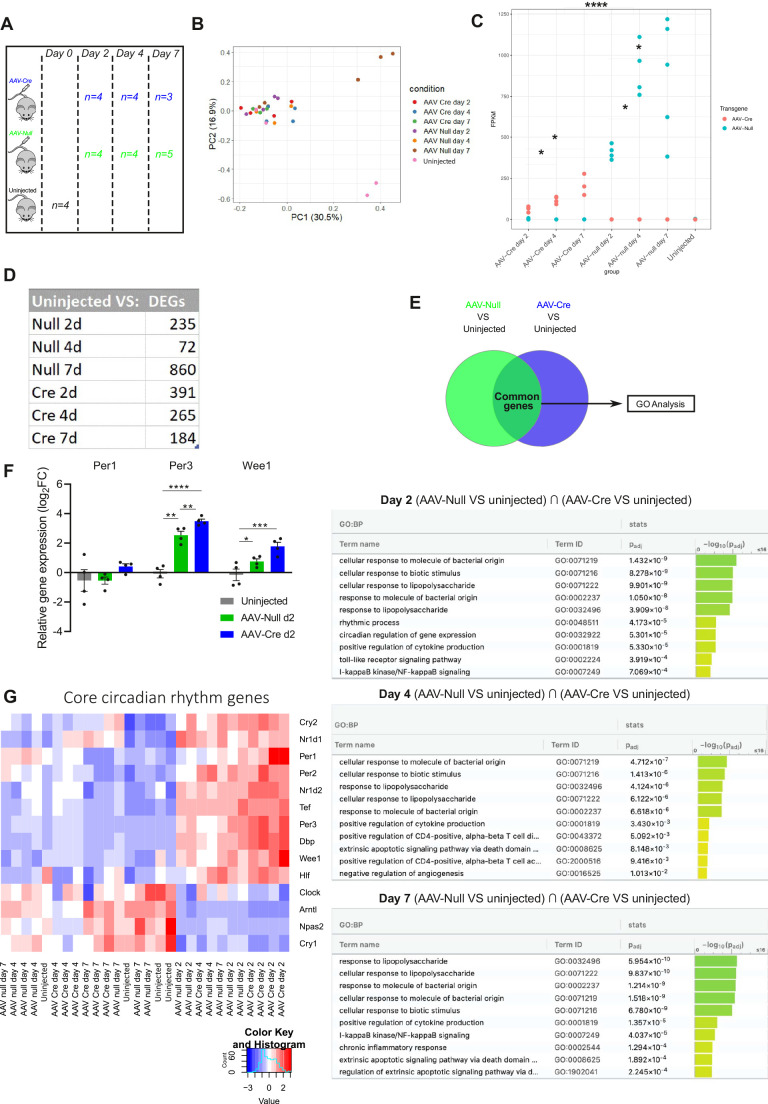
**Short-term temporal effects of AAV8-*TBG* upon the liver transcriptome.** (A) Schematic of the samples used for RNA-seq. Whole liver lysates from four uninjected, 13 AAV-Null (*n*=4 at day 2, *n*=4 at day 4 and *n*=5 at day 7 post injection) and 11 AAV-Cre (*n*=4 at day 2, *n*=4 at day 4 and *n*=3 at day 7 post injection) mice were used. (B) PCA plot of the samples used for RNA-seq. (C) Quantity of the transcripts encoded by AAV-Cre (sequence of the Cre recombinase) or AAV-Null (scrambled sequence) in the different conditions represented as fragments per kilobase of transcript per million mapped reads (FPKMs). Two-way ANOVA. ∗*P*<0.05; ∗∗∗∗*P*<0.0001. (D) Table showing the number of differentially expressed genes (DEGs) for each group compared to uninjected. FDR<0.05. (E) Gene ontology (GO) analysis comparing the differentially expressed genes shared between AAV-Null and AAV-Cre mice after each group is compared to uninjected mice (AAV-Null versus uninjected∩AAV-Cre versus uninjected) mice at day 2, 4 and 7. (F) RT-qPCR for Per1, Per3 and Wee1. Fold change expression was calculated by normalizing to the uninjected mice for each gene. *n*=4 for each group. Kruskal–Wallis test (Per1) or one-way ANOVA (Per3, Wee1). ∗*P*<0.05; ∗∗*P*<0.01; ∗∗∗*P*<0.001 and ∗∗∗∗*P*<0.0001. The bars are mean±s.e.m. (G) Unsupervised heatmap showing the differential expression of major genes involved in circadian rhythm regulation.

As a broader and unbiased assessment of the effects of AAV8-*TBG* vectors we next explored their effect on the liver transcriptome by performing RNA-seq on whole liver lysates from the AAV8-*TBG*-treated and uninjected control mice (Fig. 4A). In general, there was a strong degree of similarity among all samples by principal component analysis (PCA) (Fig. 4B). We interrogated this transcriptomics data in more detail, starting with the AAV8-*TBG* cargo in each group. Here we observed that there was a gradual increase in the number of the respective AAV8-*TBG* transcripts detected from day 2 to day 7 (Fig. 4C). Transcript number was also influenced by the specific cargo; expression of Cre transcript was lower than that of the transcript expressed by AAV-Null. Our analysis identified 235, 72, 860, 391, 265 and 184 genes that were differentially expressed between uninjected and AAV-Null day 2, AAV-Null day 4, AAV-Null day 7, AAV-Cre day 2, AAV-Cre day 4 and AAV-Cre day 7 groups, respectively (Fig. 4D). Next, we performed pathway analysis in order to identify global transcriptional changes. This revealed two broad transcriptional programmes that were altered among the different timepoints; immune response-related changes and circadian rhythm changes (Fig. 4E). This is further supported by the observation that several immune-related, genes including the principally monocyte chemoattractants Ccl2 and Cxcl9/10, are consistently differentially expressed in all groups compared to the uninjected group (Fig. S5). Notably, using this unbiased approach we did not observe any transcriptional changes associated with DDR.

Having observed prominent effects on cellular proliferation at day 2, we focused on the circadian rhythm process that was specific for this time point. First, we validated the expression of specific genes involved in circadian rhythm (Takahashi, 2017) observing similar trends of expression to those of the RNA-seq (Fig. 4F,G). Similarly to the reduced proliferation at day 2, the changes in circadian rhythm were viral-specific rather than cargo-specific; the change was observed at a specific time point regardless of the cargo (Fig. 4G). Furthermore, some of the genes involved in these networks (Wee1, Tef) have been described to regulate cell cycle (Russell and Nurse, 1987; Rowley et al., 1992; Yang et al., 2019). Overall, our transcriptomic data reveals changes in genes involved in the circadian rhythm as well as in inflammation and immunity.

## DISCUSSION

AAV8-*TBG* vectors are an established means for hepatocyte-specific manipulation of gene expression *in vivo*. In this study we show that AAV8-*TBG* vectors have both a high degree of specificity and minimal off-target effects. Therefore, they serve as a reliable and efficient experimental tool. They have a number of specific advantages over alternatives including less specific Cre expression systems, global gene knockout and even CRISPR-Cas9, which itself is widely accepted in its current form to introduce off-target Cas9 cleavage events across species and to activate the TP53 pathway signalling (Tsai et al., 2015; Enache et al., 2020; Garrood et al., 2021). To our knowledge, our study is the first one to systematically examine these effects in the liver of WT mice. We demonstrate that mouse health is generally unaffected by AAV8-*TBG* vectors as the body and liver weights exhibited the expected growth. No inflammatory response, either systemic or intrahepatic, was observed and liver histology and function remained normal. However, we have identified some subtle phenotypes that are induced by AAV8-*TBG* vectors, which should be taken into account when using this system for *in vivo* experiments in mice. These observations highlight that AAV8-*TBG* vectors are not entirely benign.

The specific targeting of hepatocytes was demonstrated by 2 reporters, RFP and GFP. Importantly, even though there were a few labelled cells in extra-hepatic tissues in our study, AAV8-*TBG* vectors showed highly specific tropism for hepatocytes as previously reported (Wang et al., 2010; Bell et al., 2011a,b). When considering phenotypic modification of hepatocytes, a low level of off-target (i.e. non-hepatocyte) recombination is unlikely to significantly affect short term studies, however it should be considered particularly when performing longer term experiments where modified cells may expand clonally.

We note differences in the labelling pattern between the 2 reporters; RFP labelling was evenly distributed across the hepatocytes, while fluorescent intensity of GFP was more heterogeneous across zones, showing preference for the pericentral hepatocytes (Zone 3), but also among cells within the same zone. We suggest that this is explained by the different mechanisms of labelling. Expression of the tdTomato gene is endogenously regulated and protein expression depends on recombination following Cre expression by the AAV8-*TBG* vector; once Cre is expressed and the LSL cassette excised, there is continuous RFP expression from the Rosa26 locus. On the other hand, GFP is expressed directly from the AAV8-*TBG* vector; therefore, its expression is predicted to vary from cell to cell depending on the quantity of viral copies delivered to each cell. The preferential labelling of pericentral hepatocytes by AAV8-*TBG*-GFP in mice has been demonstrated by others (Wang et al., 2010; Bell et al., 2011a,b) but the exact mechanism remains unclear. It has been reported that a stronger ‘pericentral tropism’ of AAV8 may underlie this (Bell et al., 2011a,b), rather than differential expression of *TBG* across the liver zones. This effect was also apparent by the zonal distribution of γH2AX positivity. Here we also observed zonal differences that are further exacerbated by the expression of Cre recombinase, further supporting a zonal preponderance for higher tropism/expression of cargo in pericentral hepatocytes.

One of the key findings of this study is the widespread DDR observed in the liver, and to a lesser extend in the splenic red pulp, as manifested by the increase in γH2AX. It has been previously shown that AAVs can, upon infection, induce DNA damage and mobilize the DNA repair machinery of the host cell in order to achieve the circular episomal form in which AAVs persist in the host cell (Schwartz et al., 2009; Cataldi and McCarty, 2013). These studies, mostly performed *in vitro*, identify DNA-PKcs as a key mediator of this process, with γH2AX being one of the DDR components involved. Our study confirms the increase of hepatocellular γH2AX in mice *in vivo* in response to AAV-Null infection. In addition, the increase in γH2AX staining in the spleen (a reported target-organ of AAV8 in other species such as the Rhesus macaque and the dog; Bell et al., 2011a,b; Greig et al., 2017), but not in the kidney, in both the AAV-Null and the AAV-Cre groups supports a vector-, rather than cargo-induced DDR. The enhanced DDR observed in the liver, but not in the spleen, of the mice injected with AAV-Cre could be explained by additional, non-specific DNA damage induced by the Cre recombinase. This enzyme can unselectively cut DNA at non-Lox sites (Loonstra et al., 2001; Janbandhu et al., 2014; Pépin et al., 2016; Lam et al., 2019). It is worth noting that, as in the case of GFP staining in the liver following AAV8-TBG-GFP administration, γH2AX showed a similar zonated staining pattern with stronger intensity in the pericentral area. This phenotype could be explained by relatively higher number of genetic copies of AAV8-*TBG* in the pericentral hepatocytes. Lastly, it is important to highlight that, in our study, despite the increase in hepatocellular γH2AX, there were no apparent changes in histology or gene expression related to DNA damage and that hepatocellular γH2AX expression is transient, reducing after 2 weeks.

The observed decrease of proliferation on day 2 in both AAV-Null and AAV-Cre indicates that this is an AAV8-*TBG* mediated effect rather than solely one mediated by the Cre recombinase as has been described by others (Loonstra et al., 2001). This reduction of proliferation is unlikely to be biologically significant in the longer term as it affects a small proportion of liver cells (a drop of approximately 0.2% of cells compared to uninjected controls). Nonetheless, it is possible that the affected liver cells are important for specific functions, so further characterisation of this phenotype should be considered depending on the experimental question being tested. One transcriptional process that was altered in AAV8-*TBG*-treated mice was the circadian rhythm, with the changes taking place on day 2. Circadian rhythm is classically viewed as an internal biological clock manifested by oscillations in gene expression, which is mainly affected by photoperiodism. The liver, however, has an additional autonomous internal clock and thus it is not entirely dependent on photoperiodism (Koronowski et al., 2019; Li et al., 2020). Our transcriptomics analysis identified several genes involved in circadian rhythm that are differentially expressed at day 2. As some of these genes have been implicated in the control of cell cycle (Matsuo et al., 2003; Zhou et al., 2018), it is possible that these transcriptional changes are related to the mild decrease in hepatic proliferation we observed at day 2.

Our transcriptomics analysis of whole liver lysates revealed that AAV8-*TBG* vectors can induce transcriptional changes in the liver. Regarding the variance observed in the PCA plots, we believe that the major driver of the principal component 1 (PC1) is inter-mouse biological variability driven by differences between inbred mouse litters. No specific pathways were responsible for this variance and in particular, after reanalysis the five outlying samples on this axis are probably littermates from a separate litter, which was relatively biologically ‘distant’ from the other litters of the study. On the other hand, PC2 (16.9% of variation) was mostly driven by the effect of the AAV vector, and particularly separated the uninjected control mice from those that received the AAV-Null vector. This is further supported by the observation that the rest of the mice cluster together on the PCA, regardless of the vector they were injected with.

The most prominent transcriptional changes identified in GO analysis are related to infection and inflammation processes and were observed in all the time points of the study. Given the viral nature of AAV8-*TBG* vectors, it is perhaps unsurprising to observe these transcriptional responses in the transduced cells. However, in our hands, this transcriptional response to infection did not result in a demonstrable immune response, as manifested by the stable proportion of hepatic immune cells at all time points. This is also supported by a similar study in Rhesus macaques where it was shown that AAV8-TBG administration induces minimal immune response in the liver (Greig et al., 2017). Nevertheless, these transcriptional changes should be considered in experiments with AAV8-*TBG*, especially when the focus of the study is related to the immune system and/or inflammation.

One limitation of our work is that we have not explored the longer term consequences of AAV8 use in WT animals. We have observed long term hepatic expression of GFP in mice at 100 days following AAV8-*TBG*-GFP administration (Barthet et al., 2021). Persistent expression of AAV8-*TBG*-driven GFP in the liver suggests persistence of AAV8-*TBG* vectors in the hepatocytes. Therefore, it would be interesting to characterise the long term effects of AAV8-*TBG* vectors in mice.

In this study we describe the short term off-target effects (i.e. effects on hepatocytes, and by extension on the whole organism, that occur by AAV8-*TBG* transduction without genetic recombination) of systemic administration of AAV8-*TBG* vectors in mice at a dose relevant for target delivery across the entire hepatocyte population. Although other studies have reported some aspects of off-target effects of AAVs, these have mostly been performed *in vitro* and only explored specific hypothesis driven effects. In our study, the use of WT C57BL/6J mice to map the AAV8-*TBG* off-target effects, both systemic and liver-specific, makes our data relevant to that of other researchers. Additionally, the unbiased transcriptomics analysis serves to generally reassure about a lack of major off-target effects within hepatocytes when using this vector system, whilst highlighting specific phenotypes that would need to be controlled for in an experiment with AAV8-*TBG* vectors. In conclusion, our data show that AAV8-*TBG* vectors are a reliable and efficient tool for hepatocyte-specific genetic manipulation with minimal off-target effects.

## MATERIALS AND METHODS

### Animal experiments

9–10 week-old male C57BL/6J WT mice (*Mus musculus*) were purchased from Charles River UK. To minimise biological variability we obtained mice from as few litters as possible. The mice were housed in cages of four to five mice/cage in a licensed, specific pathogen-free environment facility under standard conditions with a 12 h day/night cycle and *ad libitum* access to food and water. All experiments were carried out with ethical permission from the Animal Welfare and Ethical Review Body (AWERB) and in accordance with the ARRIVE guidelines (Percie du Sert et al., 2020) and the Home Office guidelines (UK licence 70/8891; protocol 2).

AAV8 experimentation was performed as previously described (Bird et al., 2018). Briefly, stock AAV8.*TBG*.PI.Cre.rBG (AAV8-*TBG*-Cre) (Addgene, 107787-AAV8) or AAV8.*TBG*.PI.Null.bGH (AAV8-*TBG*-Null) (Addgene, 105536-AAV8) (stored at −80°C) was thawed on ice, diluted in sterile PBS to achieve a working titre of 2×10^12^ genetic copies (GC)/ml and was subsequently stored at −20°C until usage. On the day of the injection the diluted AAV was thawed and each mouse was injected via the tail vein with 100 μl (2×10^11^ GC/mouse; mice in this study weighed from 22.4–29.4 g at the time of injection). This dose has been previously shown to result in genetic recombination of nearly the total hepatocyte population (Bird et al., 2018). All mice were weighed on injection day (day 0) and on their respective cull day. Changes in body weight were compared to published data for this mouse strain [The Jackson Laboratory, Body Weight Chart #000664, (accessed on 26/11/2020): https://www.jax.org/jax-mice-and-services/strain-data-sheet-pages/body-weight-chart-000664#;]. The mice were sacrificed 2, 4, 7 or 14 days post AAV8-TBG administration. Male C57BL/6J mice from the same batch and of the same age which were not injected with AAV8-TBG (uninjected controls) served as baseline controls. All mice were culled between the hours of 11:00 and 15:00 on the day of harvest. All mice were injected with BrdU (Amersham, RPN201, 250 μl per mouse) intraperitoneally 2 h before culling.

For the confirmation of tissue specificity of AAV8-TBG we used 8–12 weeks old male mice on a C57BL/6 background that were homozygotes for the R26RLSL-tdTomato allele (LSL-RFP) (Madisen et al., 2010). These mice were injected on the same day with both AAV8-*TBG*-Cre and AAV8.*TBG*.PI.eGFP.WPRE.bGH (AAV8-*TBG*-GFP) (Addgene, 105535-AAV8), both at a dose of 2×10^11^ GC/mouse as described above. These mice were culled 7 days post AAV8-TBG administration. LSL-RFP mice that were injected with 2×10^11^ GC of AAV8-*TBG*-Null and culled 7 days post injection served as controls for RFP expression.

Mice were euthanized by CO_2_ inhalation and their blood was collected immediately by cardiac puncture into EDTA-coated tubes (Sarstedt) for haematology or into lithium heparin-coated tubes (Sarstedt) for plasma biochemistry (plasma separation was performed by centrifugation at 2350 ***g*** for 10 min at room temperature, within 2 h post-harvest). Mouse weights and liver weights were recorded post mortem. The caudate lobe of the liver was immediately frozen in liquid nitrogen, the left median lobe was frozen on dry ice and the rest of the liver was fixed for 24 h in 10% neutral buffered formalin (in PBS), then changed to 70% ethanol before embedding.

As these are observational studies, power calculations were not routinely performed; however, animal numbers were chosen to reflect the expected magnitude of response taking into account the variability observed in pilot experiments and previous experience in transcriptomic analyses. For all experiments the number of biological replicates ≥3 mice per cohort.

### Haematology and plasma biochemistry analysis

Whole blood haematology was performed using an IDEXX ProCyte Dx analyzer on whole blood collected in EDTA-coated tubes (Sarstedt). Biochemical analysis of plasma was carried out using a Siemens Dimension Xpand Clinical Chemistry Analyzer following International Federation of Clinical Chemistry (IFCC) approved methods.

### Histology

4 μm tissue sections underwent antigen retrieval and then were sequentially incubated with the primary and secondary antibody. Detection was performed with 3,3′-Diaminobenzidine (DAB) and the sections were counterstained with Haematoxylin Z. Details about the antibodies and reagents can be found in Fig. S6.

Images were obtained on a Zeiss Axiovert 200 microscope using a Zeiss Axiocam MRc camera. For image analysis, stained slides were scanned using a Leica Aperio AT2 slide scanner (Leica Microsystems, UK) at 20x magnification. Quantification of blinded stained histologic sections was performed using the HALO image analysis software (V3.1.1076.363, Indica Labs). All of the slides except for the slides from day 14 were stained for a specific antibody in the same batch and processed at the same time in an autostainer, strictly keeping all incubation times (including that of DAB development) the same for all the samples. The slides from the day 14 time point were stained as a separate batch.

For multiplex immunofluorescence, 4 μm liver sections were retrieved for 25 min in Citrate buffer (pH 6) and were incubated with antibodies against GFP (Abcam, ab13970, 1:500), RFP (Rockland, 600-401-379, 1:200) and HNF4a (Santa Cruz Biotechnology, sc6556, 1:40) overnight at 4°C. This was followed by incubation with the secondary antibodies and DAPI (1 μg/μl, 0100-20, SouthernBiotech) for 1 h at room temperature. Images were obtained using a Zeiss 710 upright confocal Z6008 microscope. For the quantification, slides were scanned with the Opera Phenix scanner (Perkin Elmer) at 20x magnification. For the analysis of scanned sections, the Harmony Columbus software (Perkin Elmer) was used to create an algorithm that was subsequently used to quantify 20 random fields of view.

### RNA extraction

RNA extraction was performed using the Qiagen RNeasy kit (74104, Qiagen UK) as per the manufacturer's instructions, including the optional DNase I step. Snap frozen caudate lobe (20–30 mg) was homogenized using the Precellys Evolution homogenizer (cat. number P000062-PEVO0-A, ‘MET’ programme) in 600 µl buffer RLT/1% β-mercaptoethanol in Precellys lysing kit tubes CK14 (Precellys, P000912-LYSKO-A.0). The RNA was eluted in 30 μl RNase-free water. RNA integrity and concentration were confirmed by agarose gel electrophoresis and by using the Nanodrop 2000 (Thermo Fisher Scientific), respectively. All samples had a 260/280 ratio ≥2.

### Quantitative reverse transcription PCR (RT-qPCR)

For RT-qPCR, RNA was extracted as described above. cDNA was generated from 1μg of RNA using the Qiagen QuantiTect Reverse transcription Kit (205313, Qiagen UK) on a PTC-200 thermal cycler (MJ Research) according to the manufacturer's instructions. Omission of Reverse Transcriptase and a template-free reaction were used as negative controls. Quantitative real time PCR was performed with the SYBR Green system (204145, Qiagen UK) and using primers from Qiagen targeting *Per1* (QT00113337), *Per3* (QT00133455) or *Wee1* (QT00157696) using a QuantStudio 5 Real time PCR system (Thermo Fisher Scientific, A28140) in a 384 well plate setting (final reaction volume 10 μl per well). Each biological replicate (mouse) was run in triplicate and 18S ribosomal RNA (Rn18S, Qiagen, QT02448075) was used as a house keeping gene for normalization.

### RNA-seq analysis

Purified RNA was tested on an Agilent 2200 TapeStation (D1000 screentape) using RNA screentape and samples with a RIN value greater than seven were further processed for library preparation. RNA at a concentration of 20 ng/µl (1 µg RNA in 50 µl RNase-free water) was used to prepare libraries using the TruSeq Stranded mRNA Kit. Agilent 2200 Tapestation was used to check the quality of the libraries and Qubit (Thermo Fisher Scientific) was used to assess library quantity. The libraries were then run on the Illumina NextSeq 500 using the High Output 75 cycles kit (single end, 1×75 cycle, dual index).

Raw BCL files were converted to FASTQ files using bcl2fastq2-v2.19.1 and were aligned to the mouse genome (GRCm38) using Hisat2 (v 2.1.0) and raw counts were generated using featureCounts and the GRCm38 Gencode annotation v 84. Differential gene expression was performed using edgeR. All RNA-seq analysis graphs were generated using standard R packages. Gene ontology was performed using g:Profiler (Raudvere et al., 2019).

### Statistical analyses

Statistical analyses were performed using the Prism 9 Software (GraphPad Software, Inc.). The Shapiro-Wilk test was used to assess whether data were normally distributed. For normally distributed data, either one-way ANOVA, two-way ANOVA or the Brown-Forsythe and Welch ANOVA test was used to compare the differences between each time point and the uninjected controls. Unpaired *t*-test was used for comparisons within time points (i.e. between AAV-Null and AAV-Cre at a specific time point). The Kruskal–Wallis test was performed for non-parametric data, comparing the differences between the uninjected mice and each time point. All figures were created using the Scribus Software (v1.4.7, G.N.U. general public licence). All data points on line graphs represent mean±Standard Error of Mean (s.e.m.). In bar graphs, bars represent mean±s.e.m. and each dot represents a single mouse. In all graphs ≥4 biological replicates (mice) are used for each time point. *P*-values are: ∗*P*<0.05; ∗∗*P*<0.01; ∗∗∗*P*<0.001, and ∗∗∗∗*P*<0.0001.

## Supplementary Material

Supplementary information

## References

[BIO058678C1] Barthet, V. J. A., Brucoli, M., Ladds, M. J. G. W., Nössing, C., Kiourtis, C., Baudot, A. D., O'Prey, J., Zunino, B., Müller, M., May, S. et al. (2021). Autophagy suppresses the formation of hepatocyte-derived cancer-initiating ductular progenitor cells in the liver. *Sci. Adv.* 7, eabf9141. 10.1126/sciadv.abf9141 34088666 PMC8177709

[BIO058678C2] Bell, P., Gao, G., Haskins, M. E., Wang, L., Sleeper, M., Wang, H., Calcedo, R., Vandenberghe, L. H., Chen, S.-J., Weisse, C. et al. (2011a). Evaluation of adeno-associated viral vectors for liver-directed gene transfer in dogs. *Hum. Gene. Ther.* 22, 985-997. 10.1089/hum.2010.194 21204705 PMC3159528

[BIO058678C3] Bell, P., Wang, L., Gao, G., Haskins, M. E., Tarantal, A. F., Mccarter, R. J., Zhu, Y., Yu, H. and Wilson, J. M. (2011b). Inverse zonation of hepatocyte transduction with AAV vectors between mice and non-human primates. *Mol. Genet. Metab.* 104, 395-403. 10.1016/j.ymgme.2011.06.002 21778099 PMC3269907

[BIO058678C4] Bird, T. G., Müller, M., Boulter, L., Vincent, D. F., Ridgway, R. A., Lopez-Guadamillas, E., Lu, W.-Y., Jamieson, T., Govaere, O., Campbell, A. D. et al. (2018). TGFβ inhibition restores a regenerative response in acute liver injury by suppressing paracrine senescence. *Sci. Transl. Med.* 10, eaan1230. 10.1126/scitranslmed.aan1230 30111642 PMC6420144

[BIO058678C5] Boutin, S., Monteilhet, V., Veron, P., Leborgne, C., Benveniste, O., Montus, M. F. and Masurier, C. (2010). Prevalence of serum IgG and neutralizing factors against adeno-associated virus (AAV) types 1, 2, 5, 6, 8, and 9 in the healthy population: implications for gene therapy using AAV vectors. *Hum. Gene. Ther.* 21, 704-712. 10.1089/hum.2009.182 20095819

[BIO058678C6] Calcedo, R., Morizono, H., Wang, L., Mccarter, R., He, J., Jones, D., Batshaw, M. L. and Wilson, J. M. (2011). Adeno-associated virus antibody profiles in newborns, children, and adolescents. *Clin. Vaccine Immunol.* 18, 1586-1588. 10.1128/CVI.05107-11 21775517 PMC3165215

[BIO058678C7] Cataldi, M. P. and Mccarty, D. M. (2013). Hairpin-end conformation of adeno-associated virus genome determines interactions with DNA-repair pathways. *Gene Ther.* 20, 686-693. 10.1038/gt.2012.86 23151519 PMC3578132

[BIO058678C8] Dou, Z., Ghosh, K., Vizioli, M. G., Zhu, J., Sen, P., Wangensteen, K. J., Simithy, J., Lan, Y., Lin, Y., Zhou, Z. et al. (2017). Cytoplasmic chromatin triggers inflammation in senescence and cancer. *Nature* 550, 402-406. 10.1038/nature24050 28976970 PMC5850938

[BIO058678C9] Duan, D., Sharma, P., Yang, J., Yue, Y., Dudus, L., Zhang, Y., Fisher, K. J. and Engelhardt, J. F. (1999). Circular intermediates of recombinant adeno-associated virus have defined structural characteristics responsible for long-term episomal persistence in muscle tissue. *J. Virol.* 73, 861. 10.1128/JVI.73.1.861-861.1999 PMC110267 9765395

[BIO058678C10] Enache, O. M., Rendo, V., Abdusamad, M., Lam, D., Davison, D., Pal, S., Currimjee, N., Hess, J., Pantel, S., Nag, A. et al. (2020). Cas9 activates the p53 pathway and selects for p53-inactivating mutations. *Nat. Genet.* 52, 662-668. 10.1038/s41588-020-0623-4 32424350 PMC7343612

[BIO058678C11] Gao, F.-F., Lv, J.-W., Wang, Y., Fan, R., Li, Q., Zhang, Z. and Wei, L. (2016). Tamoxifen induces hepatotoxicity and changes to hepatocyte morphology at the early stage of endocrinotherapy in mice. *Biomed. Rep.* 4, 102-106. 10.3892/br.2015.536 26870344 PMC4726841

[BIO058678C12] Garrood, W. T., Kranjc, N., Petri, K., Kim, D. Y., Guo, J. A., Hammond, A. M., Morianou, I., Pattanayak, V., Joung, J. K., Crisanti, A. et al. (2021). Analysis of off-target effects in CRISPR-based gene drives in the human malaria mosquito. *Proc. Natl. Acad. Sci. USA* 118, e2004838117. 10.1073/pnas.2004838117 34050017 PMC8179207

[BIO058678C13] Gay, D. M., Ridgway, R. A., Müller, M., Hodder, M. C., Hedley, A., Clark, W., Leach, J. D., Jackstadt, R., Nixon, C., Huels, D. J. et al. (2019). Loss of BCL9/9l suppresses Wnt driven tumourigenesis in models that recapitulate human cancer. *Nat. Commun.* 10, 1453. 10.1038/s41467-019-09465-7 30914643 PMC6435724

[BIO058678C14] Greig, J. A., Limberis, M. P., Bell, P., Chen, S.-J., Calcedo, R., Rader, D. J. and Wilson, J. M. (2017). Non-clinical study examining AAV8.TBG.hLDLR vector-associated toxicity in chow-fed wild-type and LDLR^+/−^ Rhesus Macaques. *Hum. Gene Ther. Clin. Dev.* 28, 39-50. 10.1089/humc.2017.014 28319449 PMC5369385

[BIO058678C15] Janbandhu, V., Moik, D. and Fässler, R. (2014). Cre recombinase induces DNA damage and tetraploidy in the absence of LoxP sites. *Cell Cycle* 13, 462-470. 10.4161/cc.27271 24280829 PMC3956542

[BIO058678C16] Keeley, T. M., Horita, N. and Samuelson, L. C. (2019). Tamoxifen-induced gastric injury: effects of dose and method of administration. *Cell. Mol. Gastroenterol. Hepatol.* 8, 365-367. 10.1016/j.jcmgh.2019.06.007 31233898 PMC6713893

[BIO058678C17] Kellendonk, C., Opherk, C., Anlag, K., Schütz, G. and Tronche, F. (2000). Hepatocyte-specific expression of Cre recombinase. *Genesis* 26, 151-153. 10.1002/(SICI)1526-968X(200002)26:2<151::AID-GENE17>3.0.CO;2-E 10686615

[BIO058678C18] Koronowski, K. B., Kinouchi, K., Welz, P.-S., Smith, J. G., Zinna, V. M., Shi, J., Samad, M., Chen, S., Magnan, C. N., Kinchen, J. M. et al. (2019). Defining the Independence of the liver circadian clock. *Cell* 177, 1448-1462.e14. 10.1016/j.cell.2019.04.025 31150621 PMC6813833

[BIO058678C19] Lam, P. T., Padula, S. L., Hoang, T. V., Poth, J. E., Liu, L., Liang, C., Lefever, A. S., Wallace, L. M., Ashery-Padan, R., Riggs, P. K. et al. (2019). Considerations for the use of Cre recombinase for conditional gene deletion in the mouse lens. *Hum. Genomics* 13, 10. 10.1186/s40246-019-0192-8 30770771 PMC6377743

[BIO058678C20] Lee, H., Yoon, D. E. and Kim, K. (2020a). Genome editing methods in animal models. *Anim. Cells Syst.* 24, 8-16. 10.1080/19768354.2020.1726462 PMC7048190 32158611

[BIO058678C21] Lee, S., Zhou, P., Whyte, S. and Shin, S. (2020b). Adeno-associated virus serotype 8-mediated genetic labeling of cholangiocytes in the neonatal murine liver. *Pharmaceutics* 12, 351. 10.3390/pharmaceutics12040351 PMC7238059 32295003

[BIO058678C22] Li, H., Malani, N. and Hamilton, S. R., Schlachterman, A., Bussadori, G., Edmonson, S. E., Shah, R., Arruda, V. R., Mingozzi, F. and Wright, J. F. (2011). Assessing the potential for AAV vector genotoxicity in a murine model. *Blood* 117, 3311-3319. 10.1182/blood-2011-04-347757 21106988 PMC3069673

[BIO058678C23] Li, H., Zhang, S., Zhang, W., Chen, S., Rabearivony, A., Shi, Y., Liu, J., Corton, C. J. and Liu, C. (2020). Endogenous circadian time genes expressions in the liver of mice under constant darkness. *BMC Genomics* 21, 224 10.1186/s12864-020-6639-4 32160860 PMC7066782

[BIO058678C24] Loo, Y. M. and Gale, M. (2011). Immune signaling by RIG-I-like receptors. *Immunity* 34, 680-692. 10.1016/j.immuni.2011.05.003 21616437 PMC3177755

[BIO058678C25] Loonstra, A., Vooijs, M., Beverloo, H. B., Allak, B. A., Van Drunen, E., Kanaar, R., Berns, A. and Jonkers, J. (2001). Growth inhibition and DNA damage induced by Cre recombinase in mammalian cells. *Proc. Natl. Acad. Sci. USA* 98, 9209-9214. 10.1073/pnas.161269798 11481484 PMC55399

[BIO058678C26] Lundin, A., Porritt, M. J., Jaiswal, H., Seeliger, F., Johansson, C., Bidar, A. W., Badertscher, L., Wimberger, S., Davies, E. J., Hardaker, E. et al. (2020). Development of an ObLiGaRe Doxycycline Inducible Cas9 system for pre-clinical cancer drug discovery. *Nat. Commun.* 11, 4903. 10.1038/s41467-020-18548-9 32994412 PMC7525522

[BIO058678C27] Madisen, L., Zwingman, T. A., Sunkin, S. M., Oh, S. W., Zariwala, H. A., Gu, H., Ng, L. L., Palmiter, R. D., Hawrylycz, M. J., Jones, A. R. et al. (2010). A robust and high-throughput Cre reporting and characterization system for the whole mouse brain. *Nat. Neurosci.* 13, 133-140. 10.1038/nn.2467 20023653 PMC2840225

[BIO058678C28] Malato, Y., Naqvi, S., Schürmann, N., Ng, R., Wang, B., Zape, J., Kay, M. A., Grimm, D. and Willenbring, H. (2011). Fate tracing of mature hepatocytes in mouse liver homeostasis and regeneration. *J. Clin. Investig.* 121, 4850-4860. 10.1172/JCI59261 22105172 PMC3226005

[BIO058678C29] Matsuo, T., Yamaguchi, S., Mitsui, S., Emi, A., Shimoda, F. and Okamura, H. (2003). Control mechanism of the circadian clock for timing of cell division in vivo. *Science* 302, 255-259. 10.1126/science.1086271 12934012

[BIO058678C30] Mendell, J. R., Campbell, K., Rodino-Klapac, L., Sahenk, Z., Shilling, C., Lewis, S., Bowles, D., Gray, S., Li, C., Galloway, G. et al. (2010). Dystrophin immunity in Duchenne's muscular dystrophy. *N. Engl. J. Med.* 363, 1429-1437. 10.1056/NEJMoa1000228 20925545 PMC3014106

[BIO058678C31] Miller, D. G., Petek, L. M. and Russell, D. W. (2004). Adeno-associated virus vectors integrate at chromosome breakage sites. *Nat. Genet.* 36, 767-773. 10.1038/ng1380 15208627

[BIO058678C32] Motwani, M., Pesiridis, S. and Fitzgerald, K. A. (2019). DNA sensing by the cGAS–STING pathway in health and disease. *Nat. Rev. Genet.* 20, 657-674. 10.1038/s41576-019-0151-1 31358977

[BIO058678C33] Müller, M., Bird, T. G. and Nault, J.-C. (2020). The landscape of gene mutations in cirrhosis and hepatocellular carcinoma. *J. Hepatol.* 72, 990-1002. 10.1016/j.jhep.2020.01.019 32044402

[BIO058678C34] Nakai, H., Fuess, S., Storm, T. A., Muramatsu, S.-I., Nara, Y. and Kay, M. A. (2005). Unrestricted hepatocyte transduction with adeno-associated virus serotype 8 vectors in mice. *J. Virol.* 79, 214-224. 10.1128/JVI.79.1.214-224.2005 15596817 PMC538708

[BIO058678C47] Nathwani, A. C., Tuddenham, E.G.D., Rangarajan, S., Rosales, C., McIntosh, J., Linch, D. C., Chowdary, P., Riddell, A., Jaquilmac Pie, A., Harrington, C. et al. (2011). Adenovirus-associated virus vector–mediated gene transfer in Hemophilia B. Available at: https://www.nejm.org/doi/full/10.1056/nejmoa1108046.

[BIO058678C35] Nault, J.-C., Datta, S., Imbeaud, S., Franconi, A., Mallet, M., Couchy, G., Letouzé, E., Pilati, C., Verret, B., Blanc, J.-F. et al. (2015). Recurrent AAV2-related insertional mutagenesis in human hepatocellular carcinomas. *Nat. Genet.* 47, 1187-1193. 10.1038/ng.3389 26301494

[BIO058678C36] Pépin, G., Ferrand, J., Höning, K., Jayasekara, W. S. N., Cain, J. E., Behlke, M. A., Gough, D. J., G. Williams, B. R., Hornung, V. and Gantier, M. P. (2016). Cre-dependent DNA recombination activates a STING-dependent innate immune response. *Nucleic Acids Res.* 44, 5356-5364. 10.1093/nar/gkw405 27166376 PMC4914124

[BIO058678C37] Percie du Sert, N., Hurst, V., Ahluwalia, A., Alam, S., Avey, M. T., Baker, M., Browne, W. J., Clark, A., Cuthill, I. C., Dirnagl, U. et al. (2020). The arrive guidelines 2.0: Updated guidelines for reporting animal research. *PLoS Biol.* 18, e3000410. 10.1371/journal.pbio.3000410 32663219 PMC7360023

[BIO058678C38] Rabinowitz, J., Chan, Y. K. and Samulski, R. J. (2019). Adeno-associated Virus (AAV) versus immune response. *Viruses* 11, 102. 10.3390/v11020102 PMC6409805 30691064

[BIO058678C39] Raudvere, U., Kolberg, L., Kuzmin, I., Arak, T., Adler, P., Peterson, H. and Vilo, J. (2019). G:Profiler: a web server for functional enrichment analysis and conversions of gene lists (2019 update). *Nucleic Acids Res.* 47, W191-W198. 10.1093/nar/gkz369 31066453 PMC6602461

[BIO058678C40] Raven, A., Lu, W.-Y., Man, T. Y., Ferreira-Gonzalez, S., O'Duibhir, E., Dwyer, B. J., Thomson, J. P., Meehan, R. R., Bogorad, R., Koteliansky, V. et al. (2017). Cholangiocytes act as facultative liver stem cells during impaired hepatocyte regeneration. *Nature* 547, 350-354. 10.1038/nature23015 28700576 PMC5522613

[BIO058678C41] Rogers, G. L., Martino, A. T., Aslanidi, G. V., Jayandharan, G. R., Srivastava, A. and Herzog, R. W. (2011). Innate immune responses to AAV vectors. *Front. Microbiol.* 2, 194. 10.3389/fmicb.2011.00194 21954398 PMC3175613

[BIO058678C42] Rosenthal, N. and Brown, S. (2007). The mouse ascending: perspectives for human-disease models. *Nat. Cell Biol.* 9, 993-999. 10.1038/ncb437 17762889

[BIO058678C43] Rowley, R., Hudson, J. and Young, P. G. (1992). The wee1 protein kinase is required for radiation-induced mitotic delay. *Nature* 356, 353-355. 10.1038/356353a0 1549179

[BIO058678C44] Russell, P. and Nurse, P. (1987). Negative regulation of mitosis by wee1+, a gene encoding a protein kinase homolog. *Cell* 49, 559-567. 10.1016/0092-8674(87)90458-2 3032459

[BIO058678C45] Sauer, B. and Henderson, N. (1988). Site-specific DNA recombination in mammalian cells by the Cre recombinase of bacteriophage P1. *Proc. Natl. Acad. Sci. USA* 85, 5166-5170. 10.1073/pnas.85.14.5166 2839833 PMC281709

[BIO058678C46] Schwartz, R. A., Carson, C. T., Schuberth, C. and Weitzman, M. D. (2009). Adeno-associated virus replication induces a DNA damage response coordinated by DNA-dependent protein kinase. *J. Virol.* 83, 6269-6278. 10.1128/JVI.00318-09 19339345 PMC2687378

[BIO058678C48] Takahashi, J. S. (2017). Transcriptional architecture of the mammalian circadian clock. *Nat. Rev. Genet.* 18, 164-179. 10.1038/nrg.2016.150 27990019 PMC5501165

[BIO058678C49] Tsai, S. Q., Zheng, Z., Nguyen, N. T., Liebers, M., Topkar, V. V., Thapar, V., Wyvekens, N., Khayter, C., Iafrate, A. J., Le, L. P. et al. (2015). GUIDE-seq enables genome-wide profiling of off-target cleavage by CRISPR-Cas nucleases. *Nat. Biotechnol.* 33, 187-197. 10.1038/nbt.3117 25513782 PMC4320685

[BIO058678C50] Wang, L., Wang, H., Bell, P., Mccarter, R. J., He, J., Calcedo, R., Vandenberghe, L. H., Morizono, H., Batshaw, M. L. and Wilson, J. M. (2010). Systematic evaluation of AAV vectors for liver directed gene transfer in murine models. *Mol. Ther.* 18, 118-125. 10.1038/mt.2009.246 19861950 PMC2839210

[BIO058678C51] Wilson, J. M. (1996). Animal models of human disease for gene therapy. *J. Clin. Investig.* 97, 1138-1141. 10.1172/JCI118527 8636424 PMC507165

[BIO058678C52] Yang, J., Wang, B., Chen, H., Chen, X., Li, J., Chen, Y., Yuan, D. and Zheng, S. (2019). Thyrotroph embryonic factor is downregulated in bladder cancer and suppresses proliferation and tumorigenesis via the AKT/FOXOs signalling pathway. *Cell Prolif.* 52, e12560. (10.1111/cpr.12560 30515906 PMC6496933

[BIO058678C53] Zhou, L., Yu, Y., Sun, S., Zhang, T. and Wang, M. (2018). Cry 1 regulates the clock gene network and promotes proliferation and migration via the Akt/P53/P21 pathway in human osteosarcoma cells. *J. Cancer* 9, 2480-2491. 10.7150/jca.25213 30026846 PMC6036881

[BIO058678C54] Zincarelli, C., Soltys, S., Rengo, G. and Rabinowitz, J. E. (2008). Analysis of AAV serotypes 1-9 mediated gene expression and tropism in mice after systemic injection. *Mol. Ther.* 16, 1073-1080. 10.1038/mt.2008.76 18414476

