# Supplementary material for: Specificity and off-target effects of AAV8-TBG viral vectors for the manipulation of hepatocellular gene expression in mice
Source: Biol Open. 2021 Sep 22;10(9):bio058678. doi: 10.1242/bio.058678 (PMC8487635; doi:10.1242/bio.058678)
Supplement: Supplementary information [file biolopen-10-058678-s1.pdf]

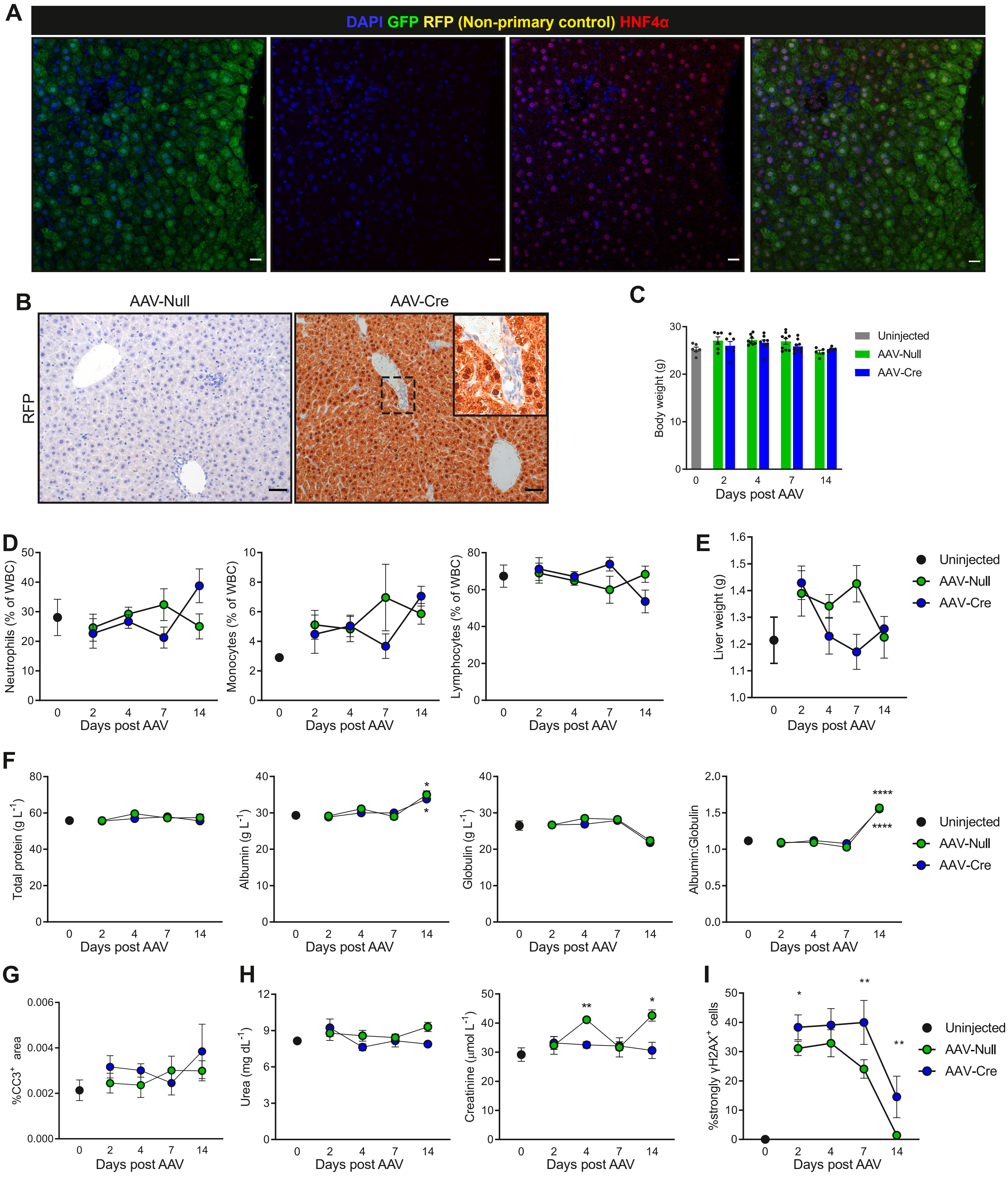

Biology Open • Supplementary information

Fig. S1. Multiplex IF controls and clinical p arameters of the mice after AAV8-TBG injection.
(A) Technical control (omission of the anti-RFP primary antibody) of the multiplex immunofluorescent stain shown in Fig.1B. Blue=DAPI, green=GFP, yellow=RFP, magenta=HNF4α (B) Chromogen-based immunohistochemistry for RFP on liver sections of LSL-tdTomato mice 7 days post AAV-Null or AAV-Cre. Image inset shows RFP- bile duct cells. (C) Body weight on the day of study initiation of the mice described in Fig. 2A. Bars are mean ± S.E.M and each dot represents one mouse. One-way ANOVA showed no statistically significant differences. (D) Relative counts of circulating neutrophils, monocytes and lymphocytes as a percentage of circulating total white blood cells of the mice described in Fig. 2A. Kruskal-Wallis test showed no statistically significant differences. (E) Liver weight of the uninjected, AAV-Null and AAV-Cre mice at the time of harvest. One-way ANOVA showed no statistically significant differences. Dots are mean ± S.E.M. (F) Plasma levels of Total Protein, Albumin, Globulin and Albumin:Globulin ratio. One-way ANOVA (Total protein) or Kruskal-Wallis test (Globulin) showed no statistically significant differences. The Kruskal-Wallis test was used for Albumin and the Brown-Forsythe and Welch ANOVA for the Albumin:Globulin ratio. P= * < 0.05, **** < 0.0001. Dots are mean ± S.E.M. (G) Quantification of Cleaved Caspase 3 (CC3) positive liver area of the mice described in 2A (n=4 for each group except day 7 and day 14 where n=5 for both AAV-Null and AAV-Cre groups). Kruskal-Wallis test showed no statistically significant differences. Dots are mean ± S.E.M. (H) Plasma levels of Urea and Creatinine. One-way ANOVA showed no statistically significant differences for Urea. The Brown-Forsythe test was used for Creatinine; P= * < 0.05. (I) Quantification of γH2AX strongly positive liver cells of the mice described in 2A (n=4 for each group except day 7 and day 14 where n=5 for both AAV-Null and AAV-Cre groups). Representative images for each time point are shown in Fig. S4. Brown-Forsythe and Welch ANOVA, P= *< 0.05, **<0.01. Dots are mean ± S.E.M. All scale bars are 50μm.

Biology Open • Supplementary information

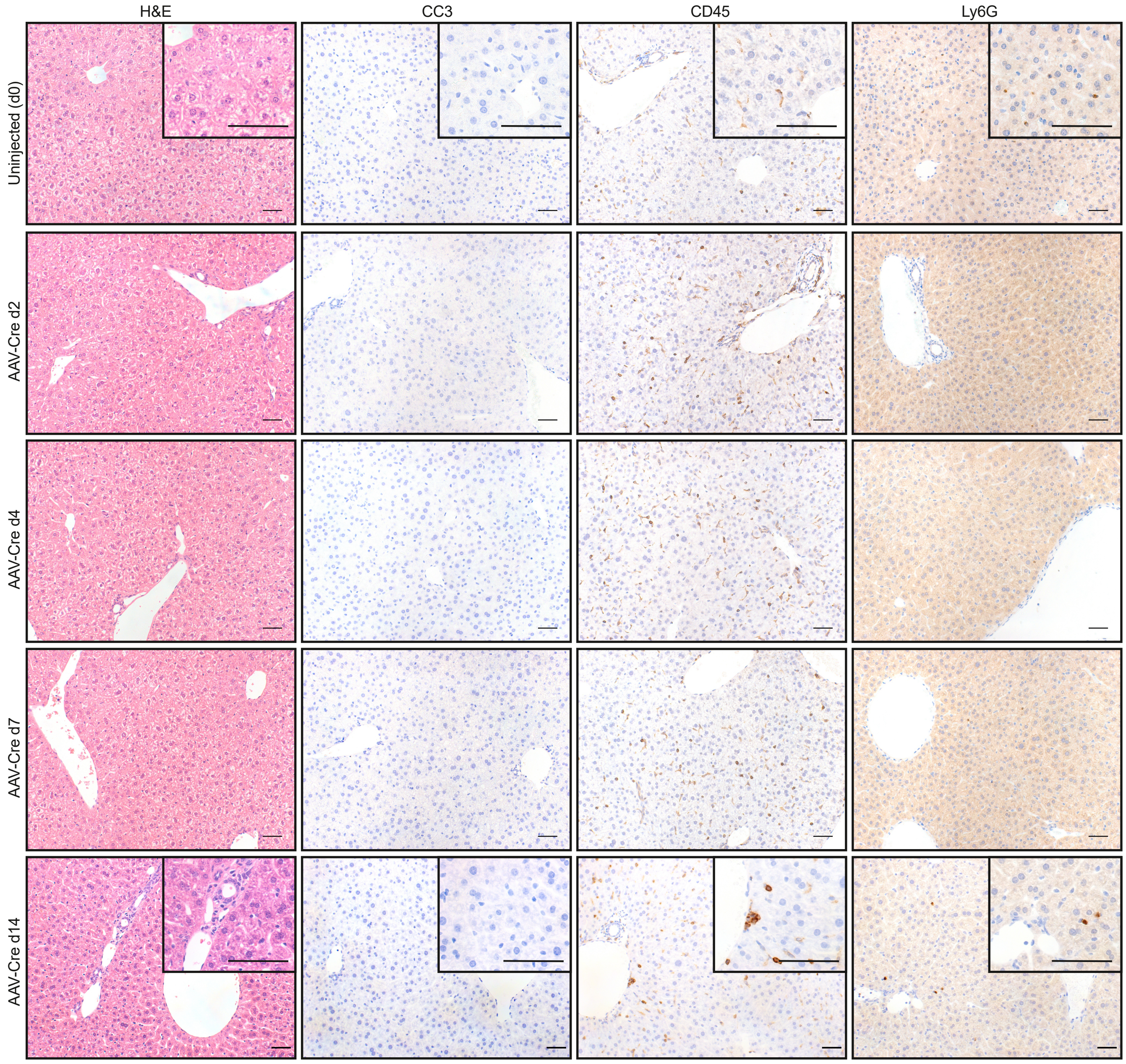

Fig. S2. Effects of AAV8-*TBG* on liver cell death and inflammation.

Representative images of H&E and immunohistochemistry for cleaved caspase-3 (CC3), CD45 and Ly6G. n=4 for each group except day 7 and day 14 where n=5. Scale bars are 50µm.

Biology Open • Supplementary information

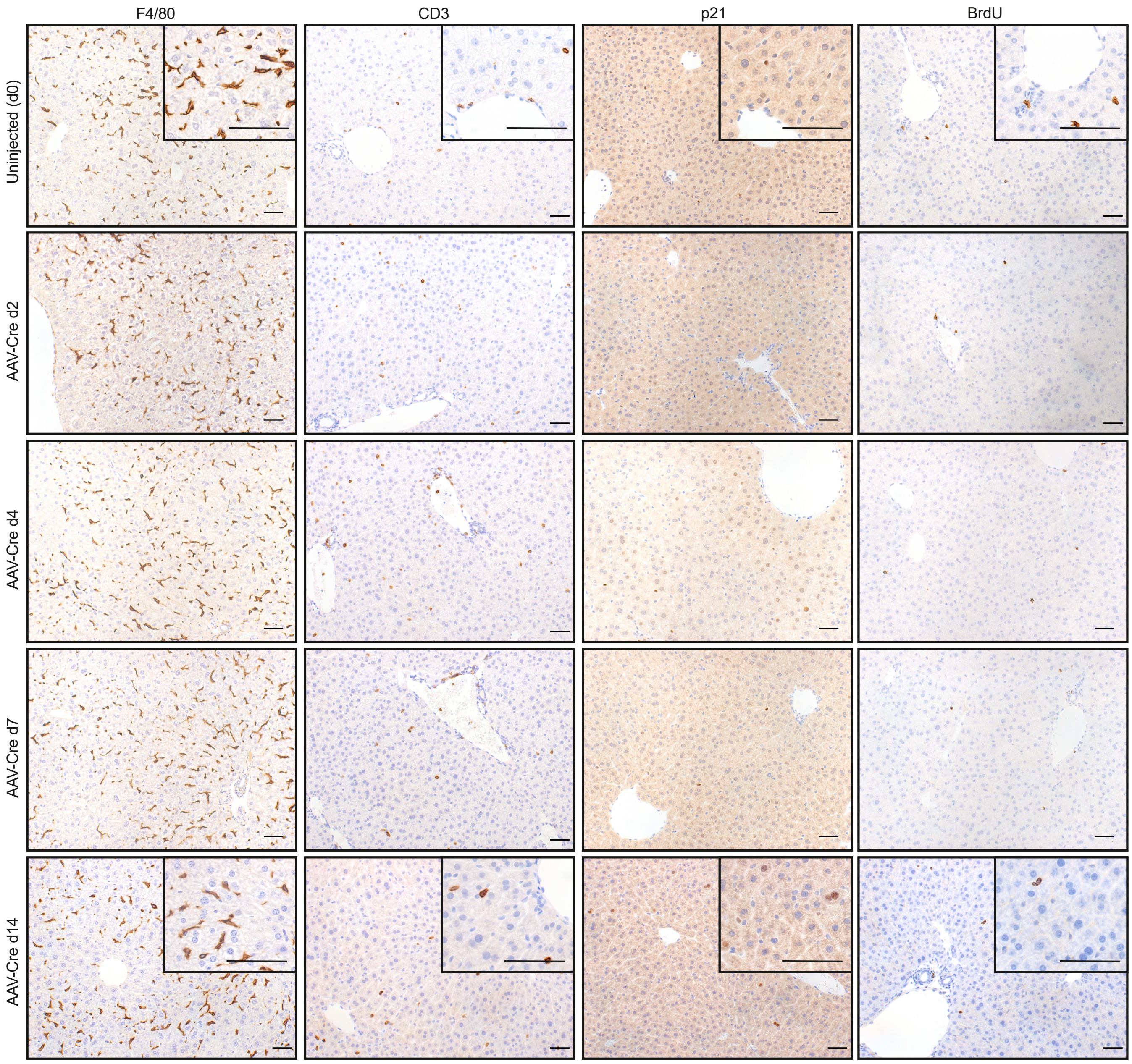

Fig. S3. Effects of AAV8-*TBG* on hepatic inflammation.

Representative photos of immunohistochemistry for F4/80, CD3, p21 and BrdU. n=4 for each group except day 7 and day 14 where n=5. Scale bars are 50µm.

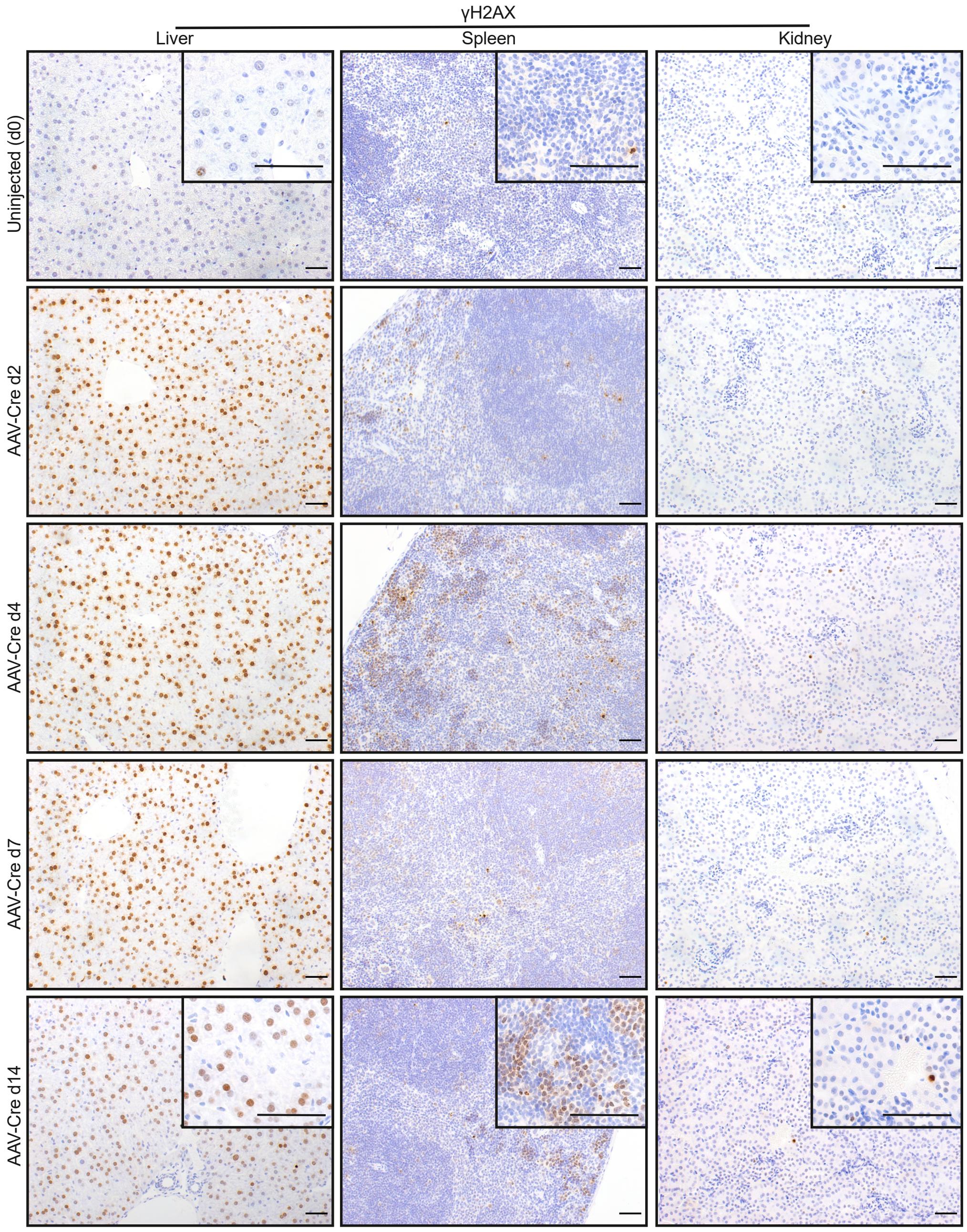

Fig. S4. Effects of AAV8-*TBG* on cell cycle and DNA damage response.

Representative photos of immunohistochemistry for γH2AX on liver, spleen and kidney sections. n=4 for each group except day 7 and day 14 where n=5. Scale bars are 50μm.

Biology Open • Supplementary information

AAV-Null

Day 2			Day 4			Day 7		
**Gene ID**	**logFC**	**FDR**	**Gene ID**	**logFC**	**FDR**	**Gene ID**	**logFC**	**FDR**
Serpine1	-7.208707	0.007621854	Serpine1	-6.684207	0.020328167	Moxd1	11.915971	0.024780481
Cxcl10	-6.513942	0.003311222	Cxcl10	-6.598603	0.007163145	Serpine1	-7.324571	0.003986209
Tnfaip3	-6.365591	0.002195043	Tnfaip3	-6.25929	0.005353276	Cxcl10	-6.651919	0.002247872
Rnd1	-5.339326	0.003004157	Ccl2	-5.158436	0.022449107	Tnfaip3	-6.296101	0.002108914
Saa3	-5.15187	0.035917137	Rnd1	-5.051423	0.010512192	Gadd45b	-5.008126	0.003986209
Gadd45b	-5.129723	0.00775671	Gadd45b	-4.84348	0.020328167	Ccl2	-4.96698	0.00652418
Ccl2	-5.093566	0.01174522	Arid5a	-4.500073	0.032592591	Rnd1	-4.54624	0.003986209
Hcar2	-5.008806	0.031685132	Icam1	-4.490287	0.032592591	Prtn3	3.96785	0.028870357
Dbp	4.749715	0.004433764	Ripk2	-4.254559	0.020328167	Irf1	-3.714232	0.004749122
Retnlg	-4.731613	0.048231374	Irf1	-3.970716	0.016109725	Syt12	3.687974	0.014593046

AAV-Cre

Day 2			Day 4			Day 7		
**Gene ID**	**logFC**	**FDR**	**Gene ID**	**logFC**	**FDR**	**Gene ID**	**logFC**	**FDR**
Cxcl10	-6.701943	1.08E-03	Tnfaip3	-6.891926	0.001194818	Serpine1	-9.499791	0.04499838
Serpine1	-6.204335	6.77E-03	Serpine1	-6.721992	0.009932564	Cxcl10	-7.080651	0.04116189
Tnfaip3	-6.106863	9.55E-04	Cxcl10	-6.71191	0.003090693	Tnfaip3	-6.515984	0.03850085
Rnd1	-5.784661	6.96E-04	Rnd1	-5.675695	0.002094203	Ccl2	-6.152802	0.04813965
Ciart	5.646927	3.76E-05	Ccl2	-5.178589	0.01241211	Atf3	-5.235571	0.04607239
Saa3	-5.350614	2.09E-02	Gadd45b	-5.104253	0.008020621	Ripk2	-4.906328	0.04499838
Dbp	5.249394	8.55E-04	Hcar2	-5.06096	0.030519716	Rnd1	-4.79663	0.04752605
Hcar2	-5.180842	1.71E-02	Saa3	-4.906788	0.042852893	Cxcl9	-4.482809	0.04752605
Ccl2	-5.164851	5.30E-03	Retnlg	-4.66692	0.044307166	Pfkfb3	-4.254039	0.04468074
Npas2	-4.541311	2.17E-05	Tlr2	-4.578716	0.026662221	Igfbp1	-3.846777	0.04228461

Fig. S5. Differentially expressed genes from the RNA-seq analysis.

Tables with the top 10 differentially expressed genes (DEGs) compared to the uninjected mice for each group.

Biology Open • Supplementary information

A

Antibody	Clone	Company	Code	Autostainer	Retreival	Dilution	Secondary Ab	Chromogen
Brdu	B44	BD Biosciences	347580	Dako Autostainer Link48	High pH TRS	1/250	Mouse EnVision	Liquid DAB
Caspase 3	ASP-175	Cell Signaling	9661	Leica Bond Rx	ER2 20 mins	1/500	Rabbit EnVision	Liquid DAB
CD3	SP7	Abcam	ab16669	Leica Bond Rx	ER2 20 mins	1/100	Rabbit Envision	Liquid DAB
CD45	N/A	Abcam	ab10558	Leica Bond Rx	ER2 20 mins	1/2500	Rabbit EnVision	Liquid DAB
F4/80	CI:A3-1	Abcam	ab6640	Leica Bond Rx	Enz1 10 mins	1/100	Rat ImmPRESS	Liquid DAB
Ly6G	IA8	BioXcell	BE0075-1	Leica Bond Rx	ER2 20 mins	1/60000	Rat ImmPRESS	Liquid DAB
p21	Hugo291	Abcam	ab107099	Leica Bond Rx	ER2 20 mins	1/150	Rat ImmPRESS	Liquid DAB
γH2AX	20E3	Cell Signaling	9718	Leica Bond Rx	ER2 10 mins	1/120	Rabbit EnVision	Liquid DAB

B

Reagent	Company	Code
Enzyme pre-treatment kit	Leica	AR9551
Epitope Retreival solution 2 (ER2)	Leica	AR9640
High pH Target Retrieval Solution (TRS)	Agilent	K8004
Liquid DAB	Agilent	K3468
Mouse EnVision	Agilent	K4001
Rabbit EnVision	Agilent	K4003
Rat ImmPRESS kit	Vector Labs	MP-7404

Fig. S6. Details of the antibodies and reagents use to perform immunohistochemistry.

(A) Table showing the methodology that was used to perform the immunohistochemistry stainings.

(B) Table showing which reagents were used for immunohistochemistry.

Biology Open • Supplementary information
